# TIMP1 may affect goat prolificacy by regulating biological function of granulosa cells

**DOI:** 10.5194/aab-65-105-2022

**Published:** 2022-03-10

**Authors:** Lei Hong, Xiang Chen, Min Zhu, Zheng Ao, Wen Tang, Zhinan Zhou

**Affiliations:** 1 Key Laboratory of Plateau Mountain Animal Genetics, Breeding and Reproduction, Ministry of Education, Guizhou University, Guiyang 550025, China; 2 Key Laboratory of Animal Genetics, Breeding and Reproduction of Guizhou province, Guizhou University, Guiyang 550025, China; 3 College of Animal Science, Guizhou University, Guiyang 550025, China

## Abstract

Tissue inhibitor of metalloproteinase 1 (TIMP1) is associated with animal reproductive processes, such as follicular growth, ovulation, luteinization, and embryo development in mammals. The purposes of this study were to explore the expression and localization of TIMP1 in the ovarian tissues and determine the effect of TIMP1 on the function of granulosa cells and the association of TIMP1 with lambing-related genes of the goats. Immunohistochemical analysis showed that TIMP1 protein was strongly expressed by granulosa cells. Enzyme-linked immunosorbent assay (ELISA) results showed that TIMP1 overexpression promoted the secretion of estradiol of granulosa cells after 12, 24, and 48 h of transfection. Moreover, in vitro experiments indicated that TIMP1 had the ability to promote the cell proliferation and elevate the transcriptional levels of four genes associated with goat prolificacy, including *BMPR-1B*, *BMP15*, *GDF9*, and *FSHB*, in granulosa cells. In conclusion, TIMP1 could be an important molecule in regulating reproductive performance of the goats by affecting estrogen secretion and cell proliferation, as well as the expression of lambing-related genes of granulosa cells in the goats.

## 1 Introduction

The reproductive performance is an important trait that affects the economic benefits of the goat industry. Lambing number is a critical indicator of reproductive traits and jointly controlled by multiple factors (Yotov et al., 2016). As a crucial organ in the reproductive system of female mammals, the ovary directly mediates the growth and maturation of oocytes. The estrogen derived from the ovary has a major impact on the fertility of goats (Zhang et al., 2015). The ovary mainly regulates the growth and development of follicle on its cortex, directly affecting lambing performance (Cui et al., 2009). The follicle is the most basic functional unit of ovarian tissue, and the growth and development of follicles are the keys to the reproduction of an animal. A complete follicle is composed of oocytes, the outer layer of granulosa cells, and the membrane. Follicles mainly rely on ovarian granulosa cells to provide nutrition and connect with oocytes to carry out information transmission. The recruitment and development of follicles, maturation and ovulation of oocytes, embryo formation, and other activities are key factors affecting reproductive traits (Anhamparuthy et al., 2009). Follicular development mainly involves the development of oocytes, granulosa cells, and follicular membranes, and granulosa cells play an important role in this process. Granulosa cells participate in the maintenance of oocyte meiotic arrest, global suppression of oocyte transcriptional activity, and the induction of oocyte meiotic and cytoplasmic maturation (Su et al., 2009). Granulosa cells provide necessary cytokines and hormones for the growth and development of oocytes. The proliferation and differentiation of granulosa cells play a crucial role in various ovarian functions, and complex autocrine and paracrine regulatory mechanisms are involved (Lu et al., 2005). Studies have found that granulosa cells of cattle, goats, and other animals mainly provide nutrients to oocytes through oocyte maturation inhibitors (OMIs). In short, the proliferation and differentiation of granulosa cells play an important role in the complete process of follicular development. The growth and development of follicles and oocytes are also inseparable from the support of ovarian granulosa cells. Therefore, the study of the function of granulosa cells contributes to the understanding of follicle development. It is also necessary to explore follicle development and the mechanism of multiple lambing in goats.

Matrix metalloproteinase inhibitors (TIMPs) are endogenous inhibitors of metalloproteinase activity (Eckfeld et al., 2019). These molecules were initially shown to play an important role in regulating the activity of matrix metalloproteinases and inhibiting the transformation of extracellular matrix (Arpino et al., 2015), including the regulation of cell proliferation, differentiation, apoptosis, etc., and play a vital role in the homeostasis of the extracellular matrix. Four members of the mammalian TIMP family are expressed in most cells, tissues, and body fluids (Zhang et al., 2019), especially in reproductive organs (Stilley et al., 2010). Studies have found that the *TIMP1* gene regulates the reproductive cycle in mice, mainly acting on ovaries and uteri (Nothnick, 2000). Furthermore, *TIMP1* deletion has been reported to lead to reproductive cycle disturbance and fertility decline in mice (Nothnick, 2003). In the ovary, the *TIMP1* gene plays a crucial role in remodeling the extracellular matrix (ECM) associated with ovulation, luteinization, and degeneration (Curry et al., 2001; Goldman et al., 2004). More importantly, *TIMP1* is also thought to be a regulator of steroid production (Nothnick et al., 2004). In sheep, the *TIMP1* gene determined embryo implantation (Hampton et al., 1995). Moreover, *TIMP1* could increase the proliferation of oviduct epithelial cells in goats (Peng et al., 2015a). Therefore, the function of *TIMP1* as an inhibitor of matrix metalloproteinases is not limited, and its important role in animal reproduction has also gradually attracted attention, which can provide new ideas for improving animal reproductive performance.

Therefore, to prove that *TIMP1* expression is associated with goat reproductive traits, we determined *TIMP1* expression and localization in goat ovarian tissue. In addition, the effect of *TIMP1* overexpression on granulosa cell proliferation, hormone secretion, and little size trait-related genes was assessed.

## 2 Materials and methods

### 2.1 Ethics statement

The animal handling procedures in this study adhered to the Animal Welfare Guidelines of the Animal Protection and Use Committee of Guizhou University, Guiyang, China (approval no. EGZU-2017T010). All animal handling procedures were performed to ensure minimal suffering.

### 2.2 Animals and tissue collection

The experimental animals came from Fuxing Animal Husbandry Co., Ltd., in Xishui County, Guizhou Province. Six healthy (2–3 years old) Qianbei Ma goats (does) with an average weight of 32 ± 0.08 kg were chosen. Ovarian tissue was collected, washed with 75 % alcohol three times, and stored in a PBS solution containing 1 % penicillin / streptomycin for cell isolation.

### 2.3 Cell culture and identification

Granulosa cells were collected from the ovaries of Qianbei Ma goats by using the follicle isolation method. Briefly, a 10-gauge needle was first used to puncture the 3–5 mm follicles to allow the follicular fluid to flow into DMEM-F/12 (Thermo Fisher Scientific, Massachusetts, USA). Subsequently, the DMEM-F/12 containing follicular fluid was transferred to a 10 mL centrifuge tube and centrifuged at 1000 rpm for 5 min. The supernatant was discarded, and DMEM-F/12 containing 10 % fetal bovine serum and 1 % antibiotics (100 IU mL ${}^{-1}$ penicillin + 50 mg mL ${}^{-1}$ streptomycin) was added to the bottom of the tube and further mixed by pipetting. The cell suspension was then transferred to a cell culture flask and incubated in a 37 ${}^{\circ }$ C cell incubator for 48 h until cell adherence reached 80 %–90 %. Immunofluorescence staining was used to identify the purity of the isolated and cultured ovarian granulosa cells.

### 2.4 Immunohistochemistry

Immunohistochemistry was performed according to a previously described protocol (Monte et al., 2019). Briefly, the ovaries were fixed with 4 % paraformaldehyde for 24 h and then embedded in paraffin. Paraffin sections (5 µ m) were attached to microscope slides, heated at 65 ${}^{\circ }$ C for 2 h, deparaffinized in xylene, and then rehydrated in a graded series of ethanol. For antigen retrieval, these sections were boiled in 0.1 M citrate buffer (pH 6.0) in a microwave oven for 15 min and then cooled to 37 ${}^{\circ }$ C. After PBS washes, endogenous peroxidase activity was blocked by incubating the sections in 3 % hydrogen peroxide for 15 min. The sections were incubated in blocking buffer (5 % BSA in PBS) for 15 min at 37 ${}^{\circ }$ C to block nonspecific binding and then incubated overnight at 4 ${}^{\circ }$ C with anti-TIMP1 polyclonal antibody ( 1:200 , Bioss, bs-0415R, Beijing, China). After PBS washes, the sections were incubated with a biotinylated secondary antibody for 2 h (37 ${}^{\circ }$ C) and HRP–streptavidin for 15 min before visualization with 3,3-diaminobenzidine tetrahydrochloride (DAB). For negative controls, the primary antibodies were replaced with PBS. Digital images were captured using a microscope.

### 2.5 RNA extraction and real-time PCR

Total RNA was extracted from goat ovary and goat granulosa cells using TRIzol reagent (Solarbio, Beijing, China). Subsequently, first-strand cDNA synthesis was carried out according to the instructions of the HiFiScript cDNA Synthesis Kit (CWBIO, Beijing, China), and the reaction products were stored in a freezer at - 20 ${}^{\circ }$ C.

For determination of the mRNA abundance of the *TIMP1*, *BMPR-1B*,* BMP15*, *GDF9*, and *FSHB* genes, a SYBR dye-based real-time quantitative PCR method was used (GenStar, Beijing, China). The primer details are listed in Table S1. RT-PCR was carried out in a CFX 9600 real-time PCR instrument (Bio-Rad, USA), and a three-step method was used (95 ${}^{\circ }$ C, 2 min, 95 ${}^{\circ }$ C, 15 s, 55 ${}^{\circ }$ C, 30 s, 72 ${}^{\circ }$ C, 30 s). Gene expression was detected in granulosa cells. Three replicates and negative controls were used each experiment. β -Actin was used for normalization. After the reactions, the data were calculated by the 2 ${}^{\Delta \Delta \text{-CT}}$ relative quantitative method (Livak et al., 2001).

### 2.6 Cloning and vector construction

The *TIMP1* coding sequence was amplified from the ovary by using Taq MasterMix (CWBIO, Beijing, China) and then inserted into a pEGFP-N3 expression vector (self repository) by using a T4 DNA Ligation Kit (Sangon Biotech, Shanghai, China). The primers used are listed in Table S1. The PCR conditions were as follows: 95 ${}^{\circ }$ C for 5 min followed by 35 cycles at 95 ${}^{\circ }$ C for 30 s, 55 ${}^{\circ }$ C for 45 s, and 72 ${}^{\circ }$ C for 30 s and a final extension of 72 ${}^{\circ }$ C for 7 min. The obtained amplicon (624 bp) was then excised with the restriction enzymes EcoR I and BamH I (TaKaRa, Dalian, China) for cloning into the pEGFP-N3 vector. The recombinant vector was called pEGFP-N3-TIMP1.

### 2.7 Cell transfection

Ovarian granulosa cells were seeded in six-well plates. When the cells grew to approximately 90 % confluence, they were cultured in antibiotic-free media containing 10 % fetal bovine serum and transfected with Lipofectamine™ 3000CD Reagent (Thermo Fisher Scientific, Massachusetts, USA). The recombinant plasmid pEGFP-N3-TIMP1 was mixed with liposomes, dropped in a crisscross pattern into the cell culture plate, and incubated in a 37 ${}^{\circ }$ and 5 % CO ${}_{2}$ incubator for 24 h.

### 2.8 Western blotting

Cells were lysed in RIPA buffer containing PMSF (Beyotime Biotechnology, Shanghai, China) for total protein extraction. The concentration was determined by BCA assays. The lysates were separated in a 12 % SDS-PAGE gel and then transferred onto a polyvinylidene fluoride membrane (Merck Millipore). After blocking, the membrane was incubated overnight at 4 ${}^{\circ }$ C with primary antibodies, namely, anti-TIMP1 ( 1:1000 , Bioss, bs-0145R, Beijing, China) and anti-GAPDH ( 1:5000 , Affinity Biosciences, AF7021, USA). The membrane was then incubated with an HRP-conjugated secondary antibody for 2 h at 37 ${}^{\circ }$ C. HRP was subsequently detected by an enhanced chemiluminescence detection system (BeyoECL Star, Beyotime Biotechnology, P0018 A, Shanghai, China). After the test, ImageJ software was used to calculate the gray value.

### 2.9 ELISA

After 12, 24, and 48 h of transfection, the culture medium was collected to detect estradiol and progesterone. Cell-free supernatants were assembled and used to evaluate estradiol (E2) and progesterone (P4) production with an enzyme-linked immunosorbent assay (ELISA) kit (Mei-Mian, Jiangsu, China). Based on the kit specifications, the absorbance at 450 nm with 50 µ L supernatants was determined using a microplate reader (Thermo Fisher Scientific, Massachusetts, USA). The corresponding concentrations of the samples were calculated using the equation from the linear regression of the obtained standard curve.

### 2.10 Cell proliferation assay

A Cell Counting Kit-8 (CCK-8) assay was carried out according to the manufacturer's instructions (Beyotime Biotechnology, Shanghai, China). Granulosa cells were transfected with pEGFP-N3-TIMP1 and seeded in 96-well plates. Further, the vessel was incubated for increasing durations (6, 12, 24, 48 h) before 10 µ L of the CCK-8 solution was added per well for 3 h. The OD value was then measured at an absorbance of 450 nm wavelength using a microplate reader (Thermo Fisher Scientific, Massachusetts, USA).

### 2.11 Statistical analysis

All data were analyzed by SPSS 19.0 and are presented as the mean ± SEM of three or five independent experiments. GraphPad Prism 5 software was used for plotting, Student's t test or one-way analysis of variance was used, and then, the least significant difference (LSD) was determined for postmortem multiple comparisons to compare the mean differences between two or more groups. Significance was accepted at the level of P<0.05 ( ${}^{*}$ P<0.05 , ${}^{**}$ P<0.01 ).

## 3 Results

### 3.1 Localization of TIMP1 in ovarian tissue

Immunohistochemistry was used to analyze the expression and localization of TIMP1 in ovarian tissues. As shown in Fig. S1, granulosa cells with brown colors were positive for TIMP1 expression, and granulosa cells with blue colors were negative controls. The results showed that TIMP1 was overexpressed in granulosa cells with brown colors, indicating that TIMP1 had a specific physiological role in ovarian granulosa cells.

### 3.2 Identification of ovarian granulosa cells

FSHR was expressed explicitly in granular cells. Therefore, FSHR can be used to identify ovarian granulosa cells. Immunofluorescence of isolated cultured granulosa cells of the ovary showed that the nuclei of all cells were blue colors (Fig. S2a). FSHR-positive cells were red colors (Fig. S2b). The cells in (a) and (b) completely overlapped (Fig. S2c), indicating that the cultured primary cells were ovarian granulosa cells of Qianbei Ma goats, with high purity, which could meet the requirements of subsequent tests.

### 3.3 Cloning and vector construction

The CDS region of the *TIMP1* gene was cloned, as shown in Fig. S3a, and the fragment length was consistent with the expected fragment size of 624 bp. The *TIMP1* gene of Qianbei Ma goats had a high degree of homology with NCBI (GenBank ID: NM_001314350.1)-logged goat sequences, with homology reaching 100 %. As shown in Fig. S3b, the recombinant plasmid pEGFP-N3-TIMP1 was double digested with the restriction enzymes EcoR I and BamH I to obtain two bands of approximately 4701 and 624 bp, indicating that the recombinant plasmid was successfully constructed.

### 3.4 TIMP1 overexpression

The recombinant plasmid pEGFP-N3-TIMP1 was transfected into ovarian granulosa cells. After 24 h, the expression of the green fluorescent protein was observed under an inverted fluorescence microscope. The cells transfected with the recombinant plasmid exhibited apparent green fluorescence (Fig. S4). After TIMP1 was overexpressed, the RT-qPCR results showed that the mRNA abundance of the TIMP1 transfection group was substantially higher than that of the blank group ( P<0.01 ). The protein level also increased in the TIMP1 transfection group ( P<0.01 ). This finding shows that the TIMP1 overexpression vector was successfully transfected.

### 3.5 Effects of the *TIMP1* gene on estradiol and progesterone secretion in granulosa cells

Detection of estradiol and progesterone was after 12, 24, and 48 h of transfection of pEGFP-N3-TIMP1 plasmid. TIMP1 overexpression gradually promoted the secretion of estradiol by granulosa cells at 12, 24, and 48 h (Fig. S5). However, TIMP1 overexpression had no significant effect on the production of progesterone (Fig. S5).

### 3.6 TIMP1 promotes the proliferation of granulosa cells

Figure S5 shows that TIMP1 overexpression promotes the proliferation of granulosa cells compared with that of the blank control group at 12 and 24 h suggesting that TIMP1 can promote the proliferation of granulosa cells.

### 3.7 TIMP1 enhances the *BMPR-1B*, *BMP15*, *GDF9*, and *FSHB* mRNA abundance

RT-qPCR were conducted to determine the effect of TIMP1 on the mRNA abundance of goat lambing trait-related genes, such as *BMPR-1B*, *BMP15*, *GDF9*, and *FSHB*. The results showed that the *BMPR-1B* mRNA abundance in the TIMP1 overexpression group was substantially higher than that in the blank control group ( P<0.01 ), and the mRNA abundance of *BMP15*, *GDF9*, and *FSHB* in the TIMP1 overexpression group was significantly higher than that in the blank control group ( P<0.05 ). The results showed that the expression of the candidate genes *BMPR-IB*, *BMP15*, *GDF9*, and *FSHB* for goat proliferation could be affected by changing the content of TIMP1 in granulosa cells (Fig. S7).

## 4 Discussion

The *TIMP1* gene belongs to the TIMP family. This gene plays an important regulatory role in the processes of many mammalian cells, including several processes related to uterine physiology, such as angiogenesis, cell differentiation, and embryo development (Pokharel et al., 2020). In the ovary, *TIMP1* is a critical gene involved in ovulation, luteinization, and degeneration. Many studies have proven that *TIMP1* plays a vital role in follicular development, ovulation, and luteal development in mammals and is closely related to animal reproductive traits. However, there is a lack of research on the Qianbei Ma goat. In this study, the immunohistochemistry results of TIMP1 expression in the ovarian granulosa cells of Qianbei Ma goats were consistent with a previous study (Peng et al., 2015b). It was speculated that the high expression of TIMP1 might have a particular influence on the proliferation and apoptosis of granulosa cells and the secretion of steroid hormones. However, the specific mechanism of how this expression regulates lambing traits is still unclear.

The ovary plays a vital role in the reproductive process of animals. This organ can secrete gonadal hormones related to reproductive estrus, follicular development, and ovulation (Kim et al., 2005). TIMP1 is mainly expressed in granulosa cells of follicles. An et al. (2012) used RT-qPCR to detect the differential expression of the *TIMP1* gene in the ovarian tissues of monotocous and polytocous goats, indicating that the *TIMP1* gene was closely related to the multiple lamb traits of goats. In this study, granulosa cells from the ovaries of Qianbei Ma goats were successfully isolated and cultured, and the eukaryotic expression vector pEGFP-N3-TIMP1 was successfully constructed and transfected into granulosa cells. Studies have shown that the estrogen signaling pathway can regulate the proliferation of granulosa cells by regulating the distribution of the cell cycle (Wada et al., 2002). With the continuous development of follicles, the volume of granulosa cells increases, and granulosa cells synthesize and secrete various factors, which have different effects on the development and atresia of follicles. When follicles ovulate, oocytes are discharged, and after ovulation, follicles differentiate into luteal tissues through the process of luteinization. When granulosa cells undergo luteinization, their morphology and function change, leading to the synthesis and secretion of large amounts of progesterone (Wang et al., 2019). Therefore, granulosa cells, as one of the crucial components of follicular development, can be used as the primary medium of oocytes to provide energy, and their morphological changes, proliferation, differentiation, cell apoptosis, cycle, and steroid hormone secretion may be involved in the regulation of the growth and development of follicles and oocytes. Que et al. (2018) found that estradiol could increase the expression of the *TIMP1* gene in rat hepatic stellate cells and promote cell proliferation. In this study, TIMP1 overexpression promoted the secretion of estradiol by ovarian granulosa at 12, 24, and 48 h, and the production of estradiol gradually increased with time. Many studies have shown that the *TIMP1* gene, as a paracrine factor or autocrine factor, may be activated through intracellular pathways, such as the ERK signaling pathway, to promote cell proliferation and inhibit cell apoptosis. For example, Dong et al. (2016) showed that the *TIMP1* gene could promote the activation and proliferation of fibroblasts through the ERK signaling pathway. In this study, the CCK-8 test was used to detect the effect of overexpression of the *TIMP1* gene on the proliferation of ovarian granulosa cells of Qianbei Ma goats. The results showed that the overexpression of the *TIMP1* gene promoted the proliferation of ovarian granulosa cells compared with that of the blank group. In addition, the *TIMP1* gene can promote the proliferation of goat oviduct epithelial cells (Peng et al., 2015a). Therefore, combined with the results of this study, it was preliminarily proven that* TIMP1* gene overexpression could promote the proliferation of ovarian granulosa cells, and the specific mechanism needs to be further studied.

Growth factors play an important role in the development and maintenance of follicles. Bone morphogenetic protein receptor 1B (*BMPR-1B*), bone morphogenetic protein 15 (*BMP15*), growth differentiation factor 9 (*GDF9*), and follicle-stimulating hormone β subunit (*FSHB*) are essential genes related to goat reproduction (Ghoreishi et al., 2019; Lan et al., 2003; Qi et al., 2019; Zi et al., 2020), and they are necessary growth factors in the ovary to initiate follicle development and regulate ovulation (Kumar et al., 2007). *BMPR-1B* affects the differentiation of granulosa cells and follicle development and can promote ovulation (Davis et al., 2005). In this study, overexpression of the *TIMP1* gene significantly increased the *BMPR-1B* mRNA abundance. Pramod et al. (2013) found that the mRNA abundance of the *BMP15* gene in the ovaries of Jintang black goats with multiple lambs was higher than that of Tibetan goats with single lambs, indicating that the* BMP15* gene in the ovaries was related to the multilamb traits of Jintang black goats. Similarly, Cui et al. (2009) also found in a comparative study of single Yunling black goats and multilamb Boer goats that the expression level of the* BMP15* gene in the ovary of Boer goats was significantly higher than that of Yunling black goats. This study found that TIMP1 overexpression could dramatically increase the mRNA abundance of the *BMP15* gene in ovarian granulosa cells. It was speculated that the *TIMP1* gene may indirectly increase the lamb number of Qianbei Ma goats by increasing the expression of the *BMP15* gene. GDF9 affects the fertility of sheep (Kona et al., 2016). This molecule is mainly secreted by oocytes and plays an important role in regulating the growth and differentiation of follicles and the development of embryos. Studies have shown that the expression of the *GDF9* gene in follicles of high-yielding Hu sheep is significantly higher than that of low-yielding Hu sheep (Xu et al., 2010). Pramod et al. (2013) found that the mRNA abundance of *BMP15* and *GDF9* genes in follicles of multilamb Black Bengal goats was significantly higher than those of single-lamb Sirohi goats, suggesting that the differential expression of these two genes was an important basis for multilamb traits. Follicular stimulating hormone (FSH) and luteinizing hormone (LH) are glycoprotein gonadotropins, which are the core hormones controlling mammalian reproduction. These hormones are synthesized and secreted by anterior pituitary basophilic cells. Zi et al. (2013) reported that the mRNA abundance of the *FSHB* gene in the pituitary gland of high-fertility Lezhi black goats during estrus was significantly higher than that of low-fertility Tibetan goats. In this study, the constructed pEGFP-N3-TIMP1 eukaryotic expression vector was transfected into ovarian granulosa cells. RT-qPCR detection showed that the mRNA abundance of the *GDF9* and *FSHB* genes in the TIMP1-overexpressing genomic cells was significantly higher than that in the blank control group. Hence, our results indicated that TIMP1 may regulate the Qianbei Ma goat lambing trait indirectly by increasing the Qianbei Ma goat ovarian granulosa cells that secrete estrogen, promoting cell proliferation, and indirectly improving the lambing trait-related gene expression, but whether it can affect the reproductive performance of goats at other levels needs further investigation.

## 5 Conclusions

In summary, TIMP1 was highly expressed in the granulosa cells of the ovary of Qianbei Ma goats, and TIMP1 overexpression promoted estradiol secretion and ovarian granulosa cell proliferation. Furthermore, TIMP1 overexpression increased the expression of *BMPR-1B*, *BMP15*, *GDF9*, and *FSHB* in ovarian granulosa cells. Our results provide a theoretical basis and experimental evidence for TIMP1 functions in goat ovarian development.

## Supplement

10.5194/aab-65-105-2022-supplement The supplement related to this article is available online at: https://doi.org/10.5194/aab-65-105-2022-supplement.

## Data Availability

The data that support this study cannot be publicly shared due to ethical or privacy reasons and may be shared upon reasonable request to the corresponding author if appropriate.

## Appendix

The supplement related to this article is available online at: https://doi.org/10.5194/aab-65-105-2022-supplement.
