# Supplementary material for: TIMP1 may affect goat prolificacy by regulating biological function of granulosa cells
Source: Arch Anim Breed. 2022 Mar 10;65(1):105–11. doi: 10.5194/aab-65-105-2022 (PMC8935209; doi:10.5194/aab-65-105-2022)
Supplement: The supplement related to this article is available online at: https://doi.org/10.5194/aab-65-105-2022-supplement. [file aab-65-105-supplement.pdf]

Supplement of Arch. Anim. Breed., 65, 105–111, 2022
https://doi.org/10.5194/aab-65-105-2022-supplement
© Author(s) 2022. CC BY 4.0 License.

Open Access
Archives Animal Breeding

Supplement of

TIMP1 may affect goat prolificacy by regulating biological function of granulosa cells

Lei Hong et al.

Correspondence to: Xiang Chen (xchen2@gzu.edu.cn)

The copyright of individual parts of the supplement might differ from the article licence.

Figure S1.

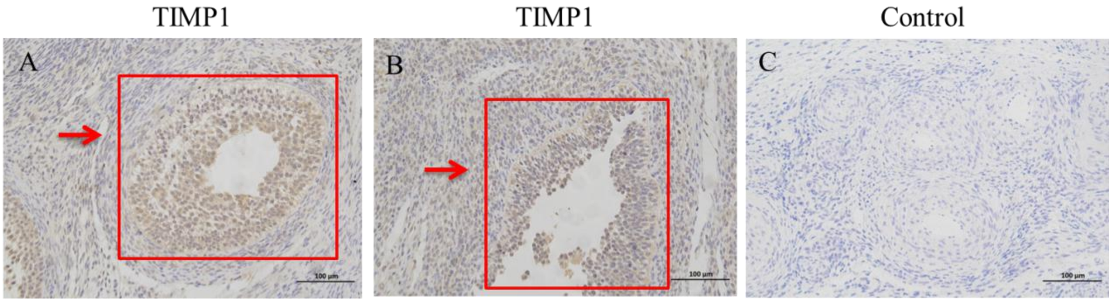

Figure S1. Immunohistochemistry of TIMP1 in ovarian tissues of Qianbei Ma goats. Ovary stained with TIMP1 (brown). A. The red box indicated by the red arrow represents a primary follicle; B. The red box indicated by the red arrow represents an atretic follicle; C. negative control; scale bar, 100 µm.

Figure S2.

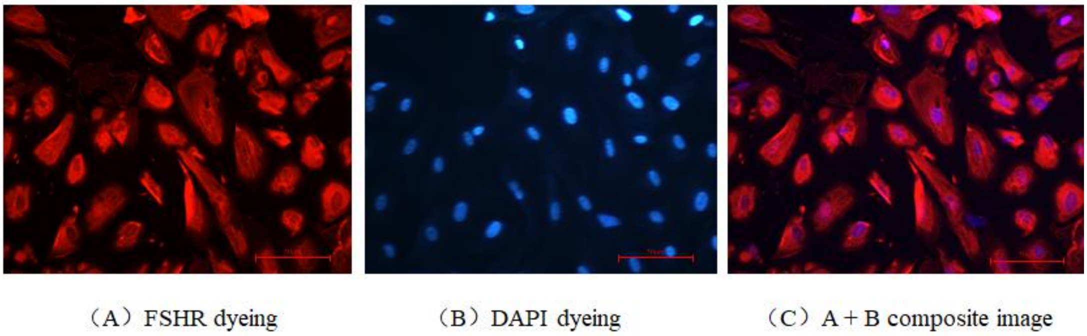

Figure S2. Immunofluorescence identification of ovarian granulosa cells. A. The cytoplasm of ovarian granulosa cells was red after FSHR (follicle stimulating hormone receptor) antibody staining. B. The nuclei of ovarian granulosa cells were blue after DAPI (4', 6-diamidino-2-phenylindole) staining. C. Nuclear and cytoplasmic staining completely overlapped. Scale bar, 100 µm.

Figure S3.

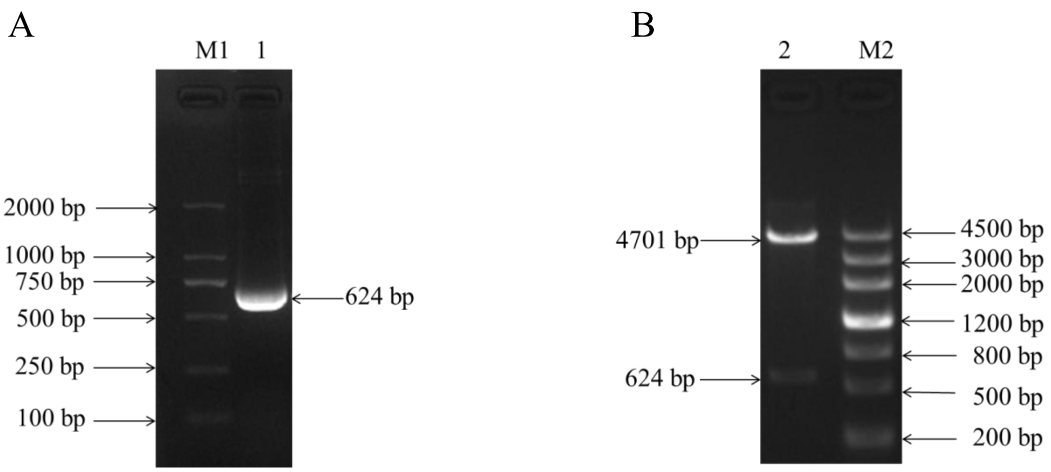

Figure S3. Construction of the recombinant vector pEGFP-N3-TIMP1. A. M1: DNA Marker 2000; 1: amplified fragment of TIMP1; B. 2: Double enzyme digestion of the recombinant vector pEGFP-N3-TIMP1, M2: Marker III.

Figure S4.

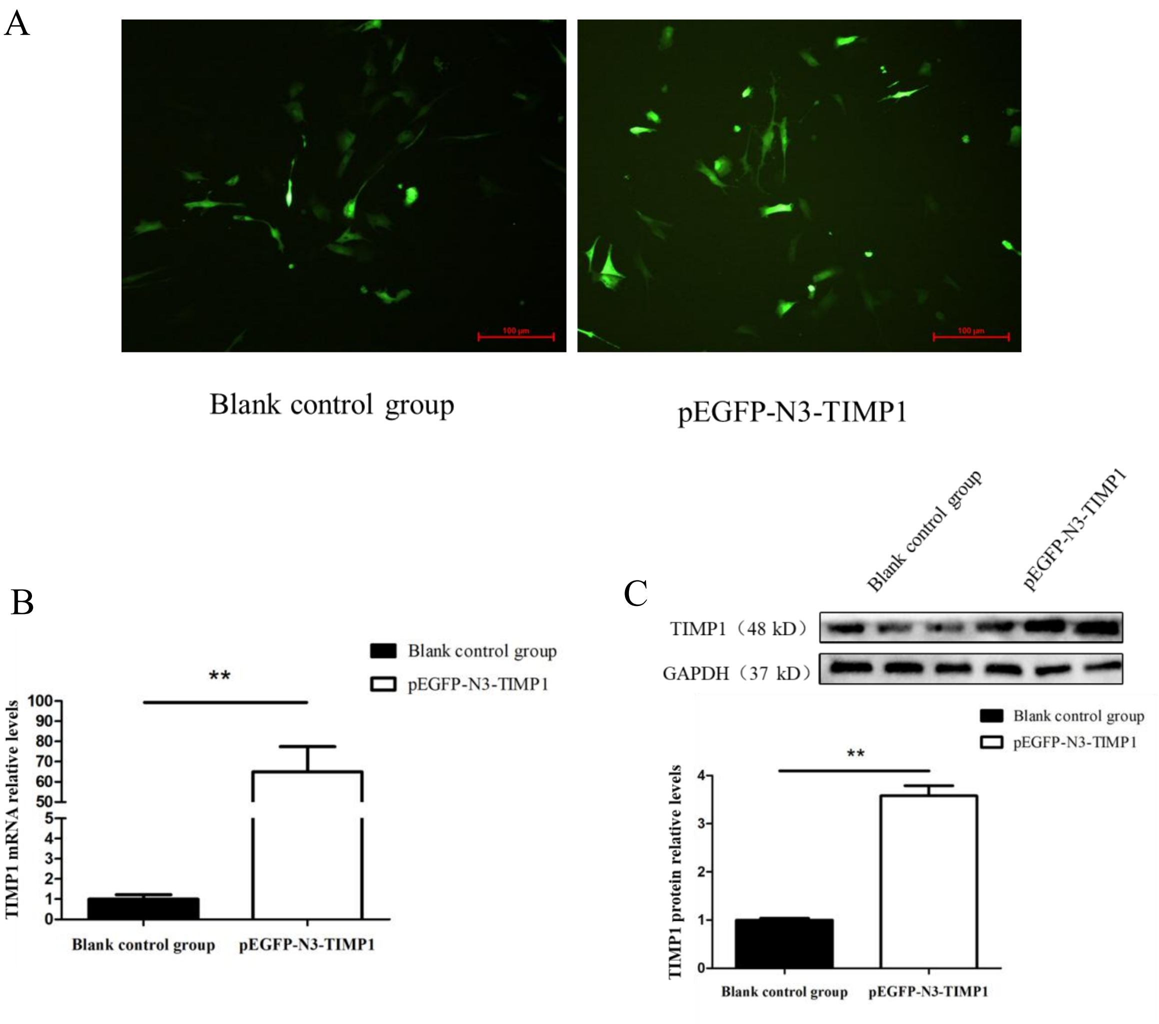

Figure S4. TIMP1 overexpression in ovarian granulosa cells. A. Green fluorescence observation. Blank control group was transfected with pEGFP-N3, and the pEGFP-N3-TIMP1 group was transfected with the recombinant plasmid; scale bar, 100 μm; B. TIMP1 mRNA abundance (n=3); C. TIMP1 protein level (n=3). ***P*<0.01.

Figure S5.

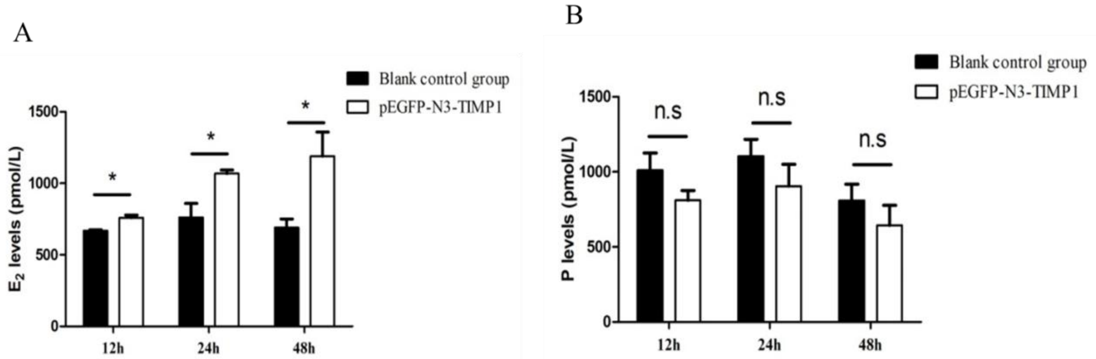

Figure S5. TIMP1 regulates estradiol (E_2) and progesterone (P) production. A. Detection of estradiol at 12 h, 24 h, and 48 h after TIMP1 overexpression. B. Detection of progesterone at 12 h, 24 h, and 48 h after TIMP1 overexpression. Error bars represent SEM (n=3), **P*<0.05, n.s. indicates no significance.

Figure S6.

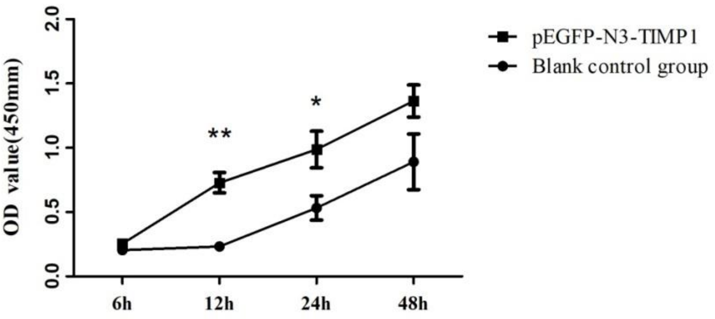

Figure S6. TIMP1 overexpression promotes ovarian granulosa cell proliferation. The OD values of the TIMP1 overexpression group were greater than those of the blank control group, as evaluated by CCK-8 assays. Error bars represent SEM (n=5), ***P*<0.01; **P*<0.05.

Figure S7.

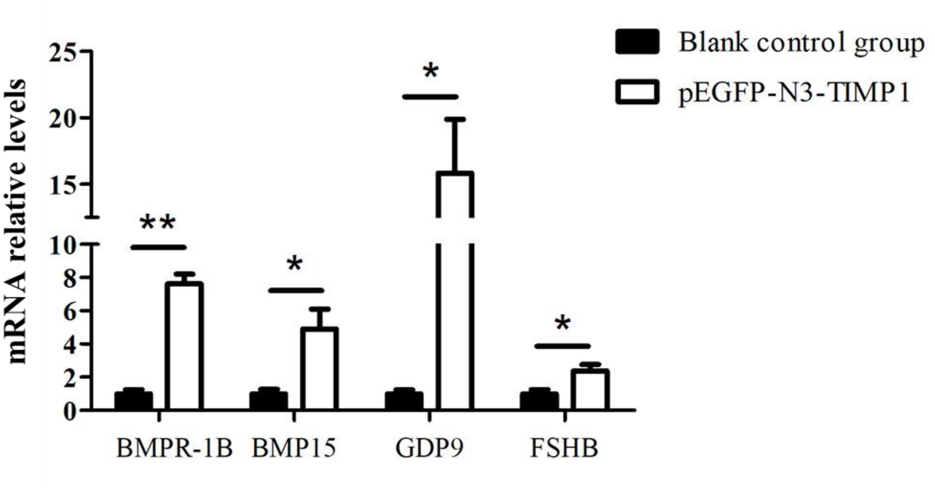

Figure S7. TIMP1 overexpression increases the expression of prolificacy candidate genes in granulosa cells. mRNA abundances of BMPR-1B, BMP15, GDF9 and FSHB in ovarian granulosa cells were determined by qPCR, error bars represent SEM (n = 3), **P*<0.05; ***P*<0.01.

Table S1. Primer sequences

Gene	Primer sequence	GenBank ID	Fragment size /bp	Tm/℃
**TIMP1**	F（EcoR I）：<br>GGAATTCCATGGCGGGGTGGAGCTGCCTCG<br>R（BamH I）：<br>CGGGATCCCGTCAGTGAATCTTTGTTTTCA	NM_001314350.1	624	59
**TIMP1**	F：GCTAATGGGTGGGCTCTGAA<br>R：GGTCAGGATGCCGTTGTTCT	NM_001314350.1	135	63
**BMP15**	F：AGAGGCTCCTGGCACATACA<br>R：GGTGAAGCTGATGGCGGTAA	NC_000023.11	143	58
**BMPR-1B**	F：CCTGGAGAATCCCTGAGAGAC<br>R：CCCCATAGCGACCTTTTCCA	NC_022298.2	136	58
**GDF9**	F：CACCGCAGAGACCAGGAGAG<br>R：TCCA GTTGTCCCACTTCAGC	NC_030814.1	99	59
**FSHB**	F：AGATGTCTGTGTGTACATGCG<br>R：GGTTGGCTCTTCTCTTGAGG	NM_001285644.1	122	58
**β-actin**	F：AGATGTGGATCAGCAAGCAG<br>R：CCAATCTCATCTCGTTTTCTG	NM_001297986.1	139	59

Note: the underlined part is the endonuclease recognition site, and the shaded part is the protective base
